# A phase II study to evaluate the safety and efficacy of topical 3% amphotericin B cream (Anfoleish) for the treatment of uncomplicated cutaneous leishmaniasis in Colombia

**DOI:** 10.1371/journal.pntd.0006653

**Published:** 2018-07-25

**Authors:** Liliana López, Iván Vélez, Claudia Asela, Claudia Cruz, Fabiana Alves, Sara Robledo, Byron Arana

**Affiliations:** 1 PECET—Programa de Estudio y Control de Enfermedades Tropicales, Facultad de Medicina, Universidad de Antioquia, Medellín, Colombia; 2 Dirección de Sanidad, DISAN, Colombian Army, Bogotá, Colombia; 3 Drugs for Neglected Diseases *initiative* (DND*i*), Geneva, Switzerland; Universidad Peruana Cayetano Heredia, PERU

## Abstract

**Background:**

Pentavalent antimonials (Sb5) are the first-line drugs for treating cutaneous leishmaniasis in Colombia; however, given problems with toxicity, compliance, availability, and cost, it is imperative to look for better therapeutic options. Intravenous amphotericin B (AmB) has been used extensively to treat visceral leishmaniasis; however, evidence on its topical use for cutaneous leishmaniasis is limited. Anfoleish is a topical formulation based on 3% AmB, which was developed following GMP standards by HUMAX and PECET. Anfoleish was shown to be safe and efficacious in animal model and in an open label study in CL patients. Hereafter we show the results of the first controlled and randomized study assessing the safety and efficacy of Anfoleish administered topically, two or three times per day for 28 days, for the treatment of non-complicated cutaneous leishmaniasis in Colombia.

**Methods:**

An open-label, randomized, non-comparative phase Ib/II clinical trial was performed. Adult volunteers with a parasitologically confirmed diagnosis of cutaneous leishmaniasis were randomly allocated to receive Anfoleish cream either 3 (TID group) or 2 (BID group) times per day for 4 weeks.

**Results:**

80 out of 105 subjects screened were included in the study. In intention to treat analysis, final cure was observed in 13 (32.5%) out of 40 subjects (IC 95% = 20.1–48) and in 12 (30%) out of 40 subjects (IC 95% = 18.1–45.5) in the BID and TID group respectively. In the per protocol analysis, cure rates were 39.4% (n = 13) (IC 95% = 24.7–56.3) and 35.3% (n = 12) (IC 95% = 21.5–52.1) in the BID and TID groups respectively. Anfoleish proved to be safe, and the few adverse events reported were local, around the area of application of the cream, and of mild intensity.

**Conclusion:**

Anfoleish showed to be a safe and well-tolerated intervention. Its efficacy results however do not support at this time continuing with its clinical development or recommending it for the treatment of CL. Additional, studies to improve its current formulation are needed before thinking in conducting additional studies in patients.

**Trial registration:**

Registered in clinicaltrials.gov NCT01845727.

## Author summary

Cutaneous leishmaniasis is a disease caused by *Leishmania* parasites and transmitted by sand flies. It is a complex disease and is underresearched disease, that depending on the parasite species, can take multiple forms, which can be less or more aggressive. It manifests as ulcers, nodules or other lesions mostly on the face and extremities. Cutaneous leishmaniasis is found across South and Central America, Africa, Europe, the Middle-East and Asia, and generally it is classified into forms of the Old World and New World. Colombia now ranks second after Brazil, with around 15,000 reported cases per year. Most cases are caused by *L*. *panamensis*, *L*. *braziliensis*, and *L*. *guyanensis*. Since the 1940’s, Pentavalent antimonials (Sb5) parenteral treatment have been the first-line of drugs for treating cutaneous leishmaniasis, however, the cost, the variable efficacy and the toxicity associated with its use, make it necessary to search for new treatments. Although there are other approved treatments for the disease, They also show issues of adverse reactions, and are used is mainly as a second option of treatment depending on the availability of these in health programs. Topical therapy of cutaneous leishmaniasis is a promising approach. Anfoleish is a cream than developed in Colombia. In this paper, we report the results of phase Ib/II, randomized trial in Colombia to assess the safety and therapeutic response of topical Anfoleish for the treatment of Cutaneous leishmaniasis.

## Introduction

Cutaneous leishmaniasis (CL) is caused by over 15 different species of the protozoan parasite *Leishmania*. CL typically begins as a papule at the site of a sand fly bite, enlarges to a nodule, and ulcerates over 1–3 months [1–3]. The exact incidence of CL is not known. An estimated 1.2 million cases/year from approximately 90 countries worldwide suffer from different forms of CL [1,4]. In the New World, ulcerative lesions are most common. Clinical syndromes of CL vary according to the infecting species and geographic distribution, but no species is uniquely associated with a particular clinical syndrome. Among the different parasites causing CL, *L*. *tropica* in the Old World and *L*. *braziliensis* in the New World are considered the most important because of the difficulty to cure, public health importance, and severity of the disease [1,5].

Colombia now ranks second after Brazil, with around 15,000 reported cases per year [4,6]. Most cases are caused by *L*. *panamensis*, *L*. *braziliensis*, and *L*. *guyanensis* [1,4]. The standard treatment is still parenteral injections of meglumine antimoniate, 20 mg/kg/day/20 days, despite its cost (USD 60–550 per course) [7], variable efficacy of between approximately 36% and 95%, and toxicity [8–10]. Miltefosine, 2.5 mg/kg/day for 28 days is also available, but is only used in cases where subjects do not respond to meglumine antimoniate or its use is contraindicated [11]. Pentamidine isothionate is sometimes available but is mainly used for subjects with lesions due to *L*. *guyanensis* [1]. There are issues with all these drugs, including mild to serious adverse reactions, compliance, availability, and cost [1,12,13].

Although Amphotericin B (AmB) has been used extensively over the last decades to treat VL, its use for treatment of CL has been limited due to its availability, the need to keep the patient hospitalized during administration, and toxicity [14,15]. Liposomal amphotericin B has also been used with good results, however its cost hampers its generalized use [16,17].

Topical therapy of CL is a promising approach. Several options have been tested, yet none have been shown to be safe, efficacious, or easy to use in the field such as would enable their adoption in all possible CL epidemiological situations. Other parameters such as frequency and duration of treatment in the presence or absence of an occlusive dressing, and whether lesions are open or closed may influence the efficacy or safety of topically applied formulations [18]. Until recently, the use of topical treatments in the New World CL patients was controversial due to the risk of further development of mucocutaneous leishmaniasis (MCL). The evidence supporting it however was assessed during the 2010 WHO Expert Committee on Leishmaniasis and it was concluded that the evidence was weak and even the use of systemic treatment does not prevent the development of MCL, hence it was recommended to include the use of topical treatment for uncomplicated CL cases due to *L*. *braziliensis*. Anfoleish is a topical formulation of a semi-solid oil in water (O / W) emulsion containing 3% AmB, developed and manufactured in accordance with Colombian regulations by Humax Pharmaceutical S.A and PECET (Programa de Estudios y Control de Enfermedades Tropicales), Medellín, Colombia. All preclinical studies were performed by PECET following international OECD pre-clinical protocols and standardized animal models.

Since there was no previous information about safety and efficacy of Anfoleish in humans, this initial study was designed as a randomized, non-comparative phase Ib/II randomzed study, aiming to evaluate the safety and efficacy of topical Anfoleish administered either two (BID) or three (TID) times per day for 28 days for the treatment of Colombian patients with CL caused by either *L*. *panamesis* or *L*. *braziliensis*.

## Methods

### Study design

This is an open-label, randomized, non-comparative, two armed exploratory study to evaluate the safety, and efficacy of two regimens of Anfoleish.

### Inclusion / exclusion criteria

Subjects who met the following criteria were included in the study: Males and females, aged ≥18 and ≤60 years old, confirmed parasitological diagnosis of CL, subjects with ≤ 3 ulcerative lesions of ≥ 0.5 cm and ≤ 3 cm (longest diameter) not located on the ear, face, close to mucosal membranes, joints, or on a location that in the opinion of the principal investigator was difficult to maintain application of the study drug topically. Criteria for exclusion were females with a positive serum pregnancy test, breast-feeding, or of a fertile age but not agreeing to take appropriate contraception during treatment period up to D45; history of clinically significant medical problems as determined by history or laboratory studies; previous use of antileishmanial drugs (within 8 weeks); or abnormal laboratory values at baseline (Hb < 10g; serum creatinine above normal level; ALT / AST 3 times above normal range).

Proof of infection was documented either through microscopic identification of amastigotes in stained lesion tissue, the demonstration of motile promastigotes in aspirate cultures, or demonstration of *Leishmania* by PCR following already published protocols [19,20].

### Population and study site

Study subjects were adult males serving in the Colombian Army attending a leishmaniasis recovery center or adults attending the PECET Clinic, both locations in Colombia.

### Intervention

Participants were randomized and allocated in a 1:1 ratio to receive either Anfoleish applied 3 times per day for 4 weeks (TID group) or Anfoleish applied twice a day for 4 weeks (BID group).

*Cream Application*: Prior to the first Anfoleish application, lesions were cleaned with soap, water, and sterile 0.9% saline, debrided, and then dried. Anfoleish was applied topically with a gloved finger to cover the whole area of the ulcer, the raised area, and rubbed into the lesion. The lesion(s) was covered and left undisturbed until the next application. At each subsequent application of the cream, the previous application and dressing was removed. Study staff members applied the cream to all lesions through Day 28. The application continued until day 28 even if the lesion had obtained 100% re-epithelialization prior to Day 28.

Rescue therapy: Meglumine antimoniate at doses of 20 mg/Sb^V^ /kg body weight per day for 20 days as recommended by Colombian Ministry of Health guidelines was provided free of charge to all subjects who met the failure criteria and those who, for whatever reason, decided to withdraw from the study.

### Follow-up and outcomes

Subjects were evaluated on a weekly basis during the treatment, at the end of treatment (day 28) and then on day 45± 5 days and on Days 90± 14 and 180± 14 to assess initial and final cure respectively.

### Endpoints

The response to treatment was evaluated clinically. The following definitions were used for each lesion:

Initial cure: Complete re-epithelialization of all ulcers and complete disappearance of the induration at Day 90 after the start of treatment.

Final Cure: Initial cure plus the absence of relapses at Day 180.

Relapse: Lesion that achieved 100% re-epithelialization by Day 90 that subsequently reopened by Day 180.

Failure was defined as <50% re-epithelialization of lesion by nominal Day 45; <100% re-epithelialization of the lesion by nominal Day 90, and relapse of the lesion at any time between D90 and D180 and an increase of ≥100% in ulcer area as compare to baseline, at any time before D90.

The percentage of re-epithelialization of the lesion(s) was calculated by comparing the size of the ulcer at baseline against the size at the follow up visit. Measures were taken after cleaning the lesion and removing the crust. Measures were done in two perpendicular directions using an electronic caliper. The area of ulceration was calculated using the area calculation for an ellipse as follows: Area = A/2*B/2*π mm^2^, where A = longest diameter of ulceration in mm; B = perpendicular to “A” diameter of ulceration in mm and π = 3.14.

### Sample size

It was calculated that a sample size of 36 subjects per treatment arm (36 TID and 36 BID) would provide a precision estimate of 15% with 95% CI, based on an anticipated cure rate at Day 90 of 70%. Accounting for 10% subjects lost during follow up, four more subjects were added resulting in sample size of 40 subjects per regimen, or 80 subjects in total.

### Pharmacokinetics (PK)

Blood samples for PK analysis were collected from the initial 30 subjects (15 subjects in each study arm) as follow: Day 1: Prior to treatment onset and at approximately 2 and 6 hours after the first Day 1 Anfoleish application; on days 14, 21 and 28, samples were obtained 2 hours after the first application of the day of Anfoleish and on day 45, at the end of the assessment visit. Plasma levels of Amphotericin B were determined using HPLC.UV methods and used to calculate the following PK parameters: Cmax, Tmax, AUC; t1/2; and λz:

It was calculated that a sample size of 36 subjects per treatment arm (36 TID and 36 BID) would provide a precision estimate of 15% with 95% CI, based on an anticipated cure rate at Day 90 of 70%. Accounting for 10% subjects lost during follow up, 4 more subjects were added resulting in an increase of the sample size to 40 for each regimen. The overall samples size was 80 subjects.

### Statistical analysis

Analyses included all randomized participants under the intention-to-treat principle. Subjects’ baseline characteristics were tabulated and analyzed for each treatment group. The efficacy of the treatments was calculated by intention to treat (ITT) and per protocol (PP). The relative risk was calculated using 2 × 2 tables. The χ^2^ test or Fisher’s exact test was used for hypothesis testing of dichotomous variables. Taking into account the distribution of the variables, a Student’s t test or Mann-Whitney U test was used for analyses of continuous data. Potential confounding factors and interactions were controlled with stratified analyses for the species of parasite responsible for the infection, number of lesions, anatomic location of the lesion, type of lesion, and geographic location where the infection occurred.

Due to the lack of information about the safety of Anfoleish when applied to Cl subjects, an interim analysis once the first 15 subjects per treatment arm had completed their 28 days treatment was planned. An ad-hoc independent safety-monitoring group reviewed safety and PK data.

### Randomization process

A list of treatments, generated randomly in blocks of six (EpiInfo, version 3.1, CDC, Atlanta, GA), was used to assign each subject to a treatment group. Numbered opaque envelopes were used to conceal the random allocation sequence. Only the study coordinator had access to the list and was in charge of assigning the treatments.

### Ethical and regulatory approvals

The protocol was approved by the bioethics committee for research on humans in the Sede de Investigación Universitaria (CBEIH-SIU) of the University of Antioquia, by the Ethics Committee of the Military Hospital of the Colombian Army and by the National Regulatory Authorities (Instituto de Vigilancia de Medicamentos y Alimentos—INVIMA), and carried out according to international norms of good clinical practice. For the participation of military, a military staff from the Sanidad Militar was invited to participate in the discussion of the project at the Universidad de Antioquia’s Ethics Committee. Approvals from Army’s Research Unit and their Institutional Ethics Committee was also obtained.

Recognizing the influences of the military command structure (in Colombia), the study consent was obtained by a study staff not affiliated to the army. The presence of army officers or any superior (in Colombia), at the time of the recruitment or during the consenting process was not allowed. Before entry into the study, investigators obtained written informed consent from all participants.

### Other procedures

After the patients signed the informed consent form for participation, a clinical form containing demographic information, data on the lesions, and a summary of the inclusion/exclusion criteria was prepared for each patient. A photographic record was also made of each lesion. Clinical samples were taken from all subjects for the parasitological confirmation of leishmaniasis.

*Leishmania* species identification was done using polymerase chain reaction–restriction fragment length polymorphism (PCR-RFLP), following established procedures [19–21]. PECET is certified by Colombian Health Authorities and quality control is conducted by the Antioquia Local Health Department.

Each application of the cream was performed by the investigators for the first 30 patients. For the additional patients, only the first application of the cream (usually in the morning) was performed by a member of the study team. Investigators observed each participant for 30 minutes after application of study drug. Lesions and surrounding skin were evaluated for pain, pruritus, erythema, and edema daily throughout treatment administration and at follow-up study visits. Clinical and laboratory evidence of side effects was determined on D7, D14, and D28 by changes from baseline in liver enzymes and serum creatinine.

Patients were not given incentives to come back for follow-up visits; patients were actively followed-up.

## Results

### Patient characteristics

**Fig 1:**
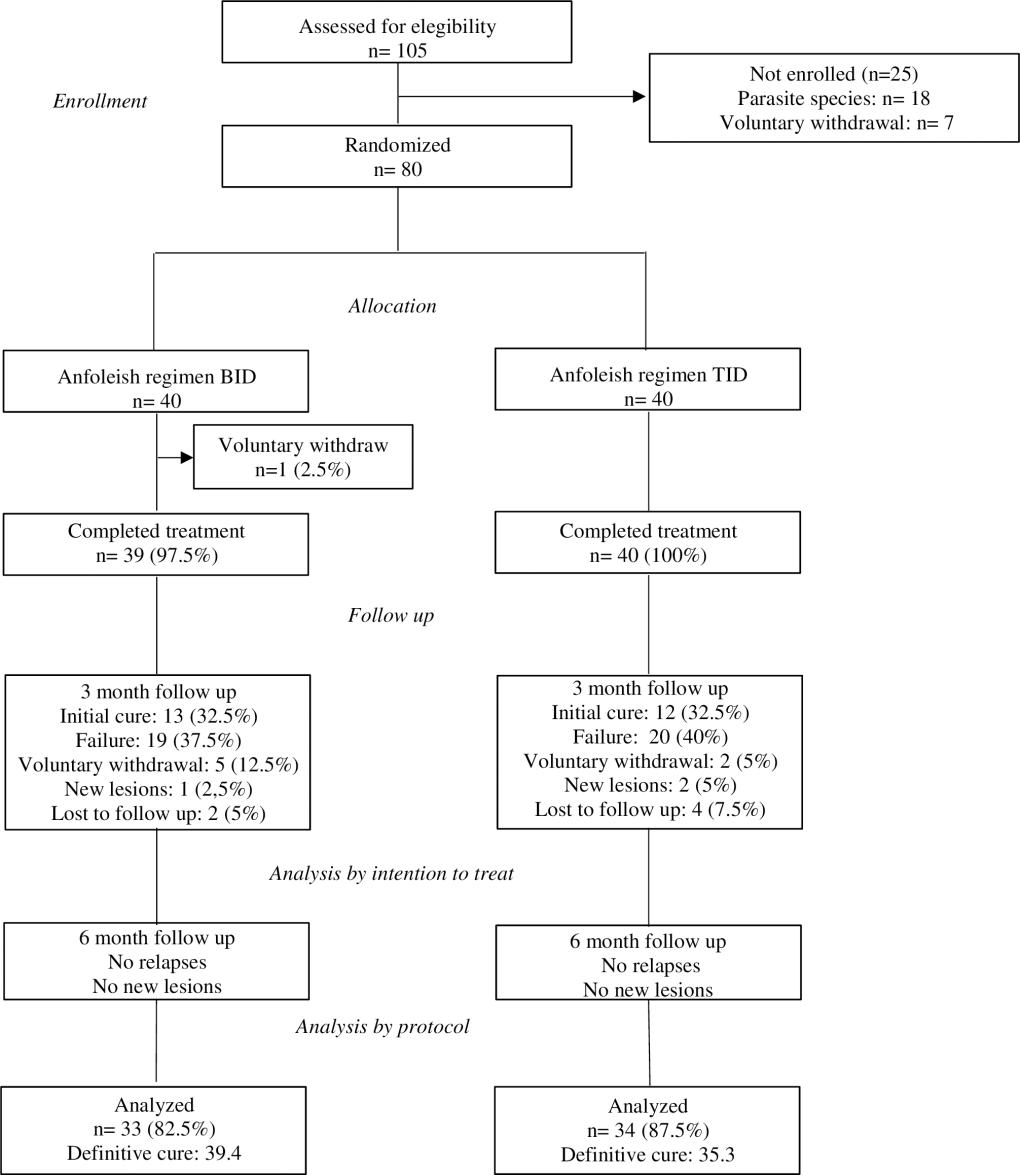
Flow diagram of the volunteer study participants.

The study was carried out between February 2014 and June 2016. Of the 105 subjects screened, 80 were enrolled and randomly assigned to receive Anfoleish either twice or three times a day for 28 days. Fig 1 shows the number of subjects per treatment group, followed and that constitute the PP and ITT population. A total of 79 subjects completed their treatment. Table 1 shows baseline subject characteristics by treatment group. Apart from lesion size, randomization successfully allocated subjects with similar characteristics, into both treatment groups. Lesions in subjects assigned to the BID group were significantly larger than the lesions of subjects assigned to the TID group (p = 0.04). All 80 subjects had their diagnosis confirmed by smear or biopsy. All but two subjects were male adults. All lesions were ulcerative, and most subjects had only one lesion (n = 72, 90%).

**Table 1:** Baseline characteristics of volunteers.

Characteristic	All	BID	TID
**Gender**			
Male (%)	78 (95%)	39 (97.5%)	39 (97.5%)
Female (%)	2 (5%)	1 (2.5%)	1 (2.5%)
**Age** (Years) Median (IQR)	24 (21–29)	24 (21–29)	24 (21–29)
***Leishmania* species**			
*L*. *braziliensis* (*Lb*) (%)	12 (15)	6 (15)	6 (15)
*L*. *panamensis* (*Lp*) (%)	66 (82.5)	33 (82.5)	33 (82.5)
*Lb / Lp* (%)^a^	2 (2.5)	1 (2.5)	1 (2.5)
**Ulcer information**			
**Size**			
Ulcer D1 (mm^2^) Median (IQR)	69.3 (32.1–215.6)	85.8 (37.3–262.5)	59.4 (28.5–174.2)
Ulcer D1 (mm^2^) Median (IQR) *Lb*	45.76 (18.6–62.1)	56.1 (38.0–64.6)	21.1 (16.8–59.3)
Ulcer D1 (mm^2^) Median (IQR) *Lp*	81.64 (35.7–244.3)	113.7 (44.1–308.4)	65.7 (33.7–196.1)
**Anatomical location**			
Head and neck (%)	12 (13.6)	6 (13.6)	6 (13.6)
Thorax (%)	7 (8)	2 (4.6)	5 (11.4)
Upper limbs (%)	55 (62.5)	30 (68.2)	25 (56.8)
Lower limbs (%)	14 (15.9)	6 (13.6)	8 (18.2)
**Number by patient**^**b**^			
One (%)	72 (90)	36 (90)	36 (90)
Two (%)	7 (8.8)	4 (10)	3 (7.5)
Three (%)	1 (1.2)	-	1 (2.5)

Me(IQR): Median and interquartile range.

^a^ In molecular identification parasites of *L*. *panamensis* and *L*. *braziliensis* species were isolated.

^b^ A total of 88 ulcers.

### Efficacy

All 80 subjects but one completed their 28 days of treatment. One patient in the BID group withdrew his consent before completing all 28 days of treatment.

In the ITT analysis, final cure was observed in 13 (32.5%) out of 40 subjects (IC 95% = 20.1–48) and in 12 (30%) out of 40 subjects (IC 95% = 18.1–45.5) in the BID and TID groups respectively. PP analysis cure rates were 39.4% (13/33) (IC 95% = 24.7–56.3) and 35.3% (12/34) (IC 95% = 21.5–52.1) in the BID and TID groups respectively. Even though the study was not designed to determine differences in cure rate by *Leishmania* species and the number of patients per group were small, it was observed that more subjects with infection due to *L*. *panamesis* were cured (35%) in comparison with subjects with infection due to *L*. *braziliensis* (8%).

The main reason for failure was an absence of initial improvement by day 45 and day 63 in 21 and 18 subjects in the BID and TID groups respectively. Seven subjects withdrew their consent to continue in the study; six were lost during the follow-up period, and three subjects were removed from the study because of the appearance of new lesions. None of the subjects declared cured at day 90 experienced a relapse of their lesion by day 180.

All subjects who fail or withdraw their consent to continue participating in the study were rescued treated as per the national treatment recommendations, using meglumine antimoniate at doses of 20 mg/kg/day for 20 days. All subjects remained under observation during their treatment and were discharged once their lesions were declared cured.

**Fig 2:**
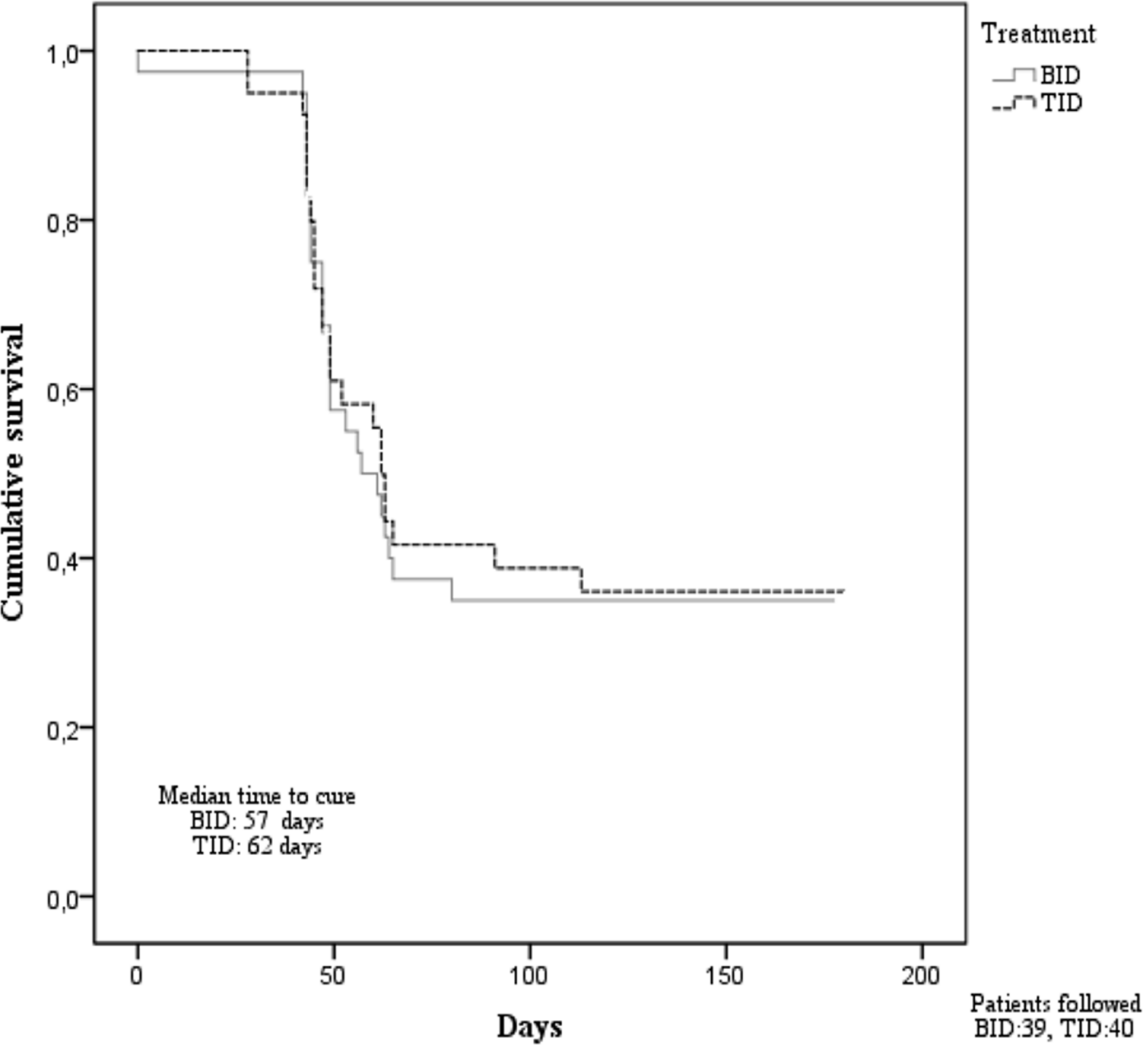
Survival analysis of time to healing of cutaneous leishmaniasis lesions.

Survival analysis, using time to heal of CL lesions as endpoint. Lesion size data time to healing of cutaneous leishmaniasis lesions at baseline (n = 80); day 45 (n = 14); day 63 (n = 3) and day 90 (n = 8) was analyzed (Fig 2).

### Safety

Only five subjects, two in the BID group and three in the TID group, reported 12 adverse events related to the cream: burning sensation, itching and rash. All were mild, affecting the area around the lesion where the cream was applied. application’s site. Three subjects experienced mild and transitory elevation of transaminases (2 subjects) or creatinine (one), all classified as of non-clinical significance, In all instances, the values returned to normal at the following control. There was one serious adverse event, varicella, which was classified as not related to the study drug. Seven subjects in each treatment group reported 36 adverse events which were classified as not related to the study drug, including flu related symptoms (7) muscle aches (6) and gastrointestinal symptoms.(10).

Regarding the PK analysis, with a minimum level detection of 4.02ng/ml, Amb was not detected in any blood plasma sample from study subjects.

## Discussion

This study was conducted according to the protocol and following the Good Clinical Practices principles. All Subjects voluntarily accepted to participate in the study and received their treatment according to the treatment arm they were randomly allocated. The follow up rate at 6 months was 92.5%. By protocol analysis, the therapeutic response of Anfoleish was 39.4% for BID group and 35.5% for TID group.

Topical treatments offer significant advantages over systemic therapy, including easier administration, fewer adverse effects, and cost-effectiveness. They are especially attractive for uncomplicated CL cases, and the use of a number of different approaches has been widely recommended, such as liquid nitrogen, local heat, or intralesional antimonials. The development of topical formulations containing AmB, the most potent anti-*Leishmania* compound identified so far, seems logical as it would reduce the toxicity of the drug compared to when it is used systemically, and also because of the scarcity of new active compounds against *Leishmania* parasites.

There are different potential explanations for the low therapeutic response to Anfoleish, including the physiochemical properties of the drug, the vehicle, the delivery system, etc. AmB has a high molecular weight and consequently transcutaneous absorption is difficult [22]. Potential problems with its penetration and absorption may have been demonstrated, given that no AmB was detected in any of the blood samples collected for pharmacokinetic analysis from the study subjects, indicating that either the levels were too low for detection by the methods used for the analysis, or that the AmB did not penetrate.

It would be interesting to consider the development of new formulations of Anfoleish by evaluating other concentrations of the compound and/or modifying the vehicle to improve its absorption and retention within the tissue [22–25].

Although the therapeutic response was not as anticipated, the absence of a control group in the study makes it difficult to estimate the actual performance of the cream. The exploratory analysis performed in the group of patients who failed revealed a median decrease in the area of lesions in at least 25% of these patients (Table 2). This finding might suggest some activity of Anfoleish, another option that might worth exploring is the use of Anfoleish in combination with other treatments to determine if the overall efficacy can be improved. There were no differences in the therapeutic response observed between BID and TID groups, which might lead us to believe that in the future two applications per day might be sufficient. Since the vast majority of patients had infections due to *L*. *panamensis*, it was impossible to perform an analysis of cure rates by species of *Leishmania*. Of note, the cure rate achieved by Anfoleish in subjected with lesions due to L. braziliensis (8.3%) was close to the rate of spontaneous cure (6.4%) reported using placebo interventions in patients with the same infecting specie [26].

**Table 2:** Change in the ulcer area during follow-up respect onset treatment in who failed (exploratory analysis).

Treatment group	BID				TID			
Ulcer area^a^	Ulcer 1		Ulcer 2		Ulcer 1		Ulcer 2	
	Me (IQR)^b^	P^c^	Me (IQR)	p	Me (IQR)	p	Me (IQR)	p
Treatment onset	85.7 (44.1–186.6)	-	87.4 (34.13–137.4)	-	50.1 (26.4–142.1)	-	77.5 (14.9–83.4)	-
Day 7	104.6 (49.2–195.8)	0.5	63.4 (47.9–123.7)	0.5	61.9 (28.4–113.5)	0.8	46.7 (6.56–95.1)	0.6
Day 14	107.5 (44–207.1)	0.7	29.6 (7–147.4)	0.3	56.9 (19.7–140.3)	0.9	42.6 (4.5–88.5)	0.3
Day 21	86 (39–271)	0.9	3.1 (0–91)	0.04	62.8 (14.5–189.7)	0.8	58.1 (4–91.6)	0.3
Day 28	107.7 (20.2–182.8)	0.9	0 (0–62.7)	0.04	59.5 (9.5–261.6)	0.4	0 (0–216.3)	1
Day 45	89.8 (11.8–238.6)	0.9	6.1 (0–177.5)	0.5	111.7 (30.6–351.1)	0.05	1.5 (0–216.3)	1

^a^ Only one volunteer (TID group) had three ulcers and was not included in the analysis.

^b^ Me(IQR): Median and interquartile range.

^c^ Wilcoxon test.

In terms of safety, Anfoleish proved to be safe, and the few adverse events reported were local and of mild intensity (mainly burning, pruritus, rash, and erythema) in the zone of application of the cream.

**In conclusion:** Although Anfoleish showed to be a safe and well tolerated option, its efficacy results do not support continue with its clinical development as a therapeutic option for CL, Additional formulation studies are needed to improve its current presentation before conducting more clinical studies.

## Supporting information

S1 Checklist Consolidated Standards of Reporting Trials (CONSORT) checklist. (PDF)

## References

[1] World Health Organization (WHO) (2010) Control of the leishmaniasis. 202 p.

[2] de Vries HJ, Reedijk SH, Schallig HD (2015) Cutaneous leishmaniasis: recent developments in diagnosis and management. Am J Clin Dermatol 16: 99–109. 10.1007/s40257-015-0114-z 25687688 PMC4363483

[3] Reithinger R, Dujardin JC, Louzir H, Pirmez C, Alexander B, et al (2007) Cutaneous leishmaniasis. Lancet Infect Dis 7: 581–596. 10.1016/S1473-3099(07)70209-8 17714672

[4] Alvar J, Velez ID, Bern C, Herrero M, Desjeux P, et al (2012) Leishmaniasis worldwide and global estimates of its incidence. PLoS One 7: e35671 10.1371/journal.pone.0035671 22693548 PMC3365071

[5] Silva NS, Muniz VD (2009) [Epidemiology of American tegumentary leishmaniasis in the State of Acre, Brazilian Amazon]. Cad Saude Publica 25: 1325–1336. 19503963 10.1590/s0102-311x2009000600015

[6] Velez ID, Carrillo LM, Lopez L, Rodriguez E, Robledo SM (2012) An epidemic outbreak of canine cutaneous leishmaniasis in Colombia caused by Leishmania braziliensis and Leishmania panamensis. Am J Trop Med Hyg 86: 807–811. 10.4269/ajtmh.2012.11-0408 22556078 PMC3335684

[7] Cardona-Arias JA, Lopez-Carvajal L, Tamayo Plata MP, Dario-Velez I (2017) Cost-effectiveness analysis of thermotherapy versus pentavalent antimonials for the treatment of cutaneous leishmaniasis. J Evid Based Med. 10.1111/jebm.12245 28276641

[8] Martinez S, Marr JJ (1992) Allopurinol in the treatment of American cutaneous leishmaniasis. N Engl J Med 326: 741–744. 10.1056/NEJM199203123261105 1738379

[9] Yazdanpanah MJ, Banihashemi M, Pezeshkpoor F, Khajedaluee M, Famili S, et al (2011) Comparison of oral zinc sulfate with systemic meglumine antimoniate in the treatment of cutaneous leishmaniasis. Dermatol Res Pract 2011: 269515 10.1155/2011/269515 21747837 PMC3130957

[10] Lopez-Jaramillo P, Rincon MY, Garcia RG, Silva SY, Smith E, et al (2010) A controlled, randomized-blinded clinical trial to assess the efficacy of a nitric oxide releasing patch in the treatment of cutaneous leishmaniasis by Leishmania (V.) panamensis. Am J Trop Med Hyg 83: 97–101. 10.4269/ajtmh.2010.09-0287 20595484 PMC2912582

[11] Velez I, Lopez L, Sanchez X, Mestra L, Rojas C, et al (2010) Efficacy of miltefosine for the treatment of American cutaneous leishmaniasis. Am J Trop Med Hyg 83: 351–356. 10.4269/ajtmh.2010.10-0060 20682881 PMC2911184

[12] Copeland NK, Aronson NE (2015) Leishmaniasis: treatment updates and clinical practice guidelines review. Curr Opin Infect Dis 28: 426–437. 10.1097/QCO.0000000000000194 26312442

[13] Lee SA, Hasbun R (2003) Therapy of cutaneous leishmaniasis. Int J Infect Dis 7: 86–93. 12839708 10.1016/s1201-9712(03)90002-6

[14] Murray HW (2012) Leishmaniasis in the United States: treatment in 2012. Am J Trop Med Hyg 86: 434–440. 10.4269/ajtmh.2012.11-0682 22403313 PMC3284358

[15] Neves LO, Talhari AC, Gadelha EP, Silva Junior RM, Guerra JA, et al (2011) A randomized clinical trial comparing meglumine antimoniate, pentamidine and amphotericin B for the treatment of cutaneous leishmaniasis by Leishmania guyanensis. An Bras Dermatol 86: 1092–1101. 22281895 10.1590/s0365-05962011000600005

[16] Solomon M, Pavlotsky F, Leshem E, Ephros M, Trau H, et al (2011) Liposomal amphotericin B treatment of cutaneous leishmaniasis due to Leishmania tropica. J Eur Acad Dermatol Venereol 25: 973–977. 10.1111/j.1468-3083.2010.03908.x 21129042

[17] Solomon M, Pavlotzky F, Barzilai A, Schwartz E (2013) Liposomal amphotericin B in comparison to sodium stibogluconate for Leishmania braziliensis cutaneous leishmaniasis in travelers. J Am Acad Dermatol 68: 284–289. 10.1016/j.jaad.2012.06.014 22858005

[18] Lecoeur H, Buffet P, Morizot G, Goyard S, Guigon G, et al (2007) Optimization of topical therapy for Leishmania major localized cutaneous leishmaniasis using a reliable C57BL/6 Model. PLoS Negl Trop Dis 1: e34 10.1371/journal.pntd.0000034 18060082 PMC2100369

[19] Montalvo AM, Fraga J, Tirado D, Blandon G, Alba A, et al (2017) Detection and identification of Leishmania spp.: application of two hsp70-based PCR-RFLP protocols to clinical samples from the New World. Parasitol Res 116: 1843–1848. 10.1007/s00436-017-5454-6 28573463

[20] Montalvo AM, Fraga J, Maes I, Dujardin JC, Van der Auwera G (2012) Three new sensitive and specific heat-shock protein 70 PCRs for global Leishmania species identification. Eur J Clin Microbiol Infect Dis 31: 1453–1461. 10.1007/s10096-011-1463-z 22083340

[21] Ramirez JR, Agudelo S, Muskus C, Alzate JF, Berberich C, et al (2000) Diagnosis of cutaneous leishmaniasis in Colombia: the sampling site within lesions influences the sensitivity of parasitologic diagnosis. J Clin Microbiol 38: 3768–3773. 11015400 10.1128/jcm.38.10.3768-3773.2000 PMC87473

[22] Perez AP, Altube MJ, Schilrreff P, Apezteguia G, Celes FS, et al (2016) Topical amphotericin B in ultradeformable liposomes: Formulation, skin penetration study, antifungal and antileishmanial activity in vitro. Colloids Surf B Biointerfaces 139: 190–198. 10.1016/j.colsurfb.2015.12.003 26709977

[23] Butani D, Yewale C, Misra A (2016) Topical Amphotericin B solid lipid nanoparticles: Design and development. Colloids Surf B Biointerfaces 139: 17–24. 10.1016/j.colsurfb.2015.07.032 26700229

[24] Fang JY, Fang CL, Liu CH, Su YH (2008) Lipid nanoparticles as vehicles for topical psoralen delivery: solid lipid nanoparticles (SLN) versus nanostructured lipid carriers (NLC). Eur J Pharm Biopharm 70: 633–640. 10.1016/j.ejpb.2008.05.008 18577447

[25] Gutierrez V, Seabra AB, Reguera RM, Khandare J, Calderon M (2016) New approaches from nanomedicine for treating leishmaniasis. Chem Soc Rev 45: 152–168. 10.1039/c5cs00674k 26487097

[26] Cota GF, de Sousa MR, Fereguetti TO, Saleme PS, Alvarisa TK, et al (2016) The Cure Rate after Placebo or No Therapy in American Cutaneous Leishmaniasis: A Systematic Review and Meta-Analysis. PLoS One 11: e0149697 10.1371/journal.pone.0149697 26894430 PMC4760744

